# Supplementary material for: In Search of Authenticity Biomarkers in Food Supplements Containing Sea Buckthorn: A Metabolomics Approach
Source: Foods. 2023 Dec 15;12(24):4493. doi: 10.3390/foods12244493 (PMC10742966; doi:10.3390/foods12244493)
Supplement: Supplementary file 1 [file foods-12-04493-s001.zip › Supplementary Figure S1.pdf]

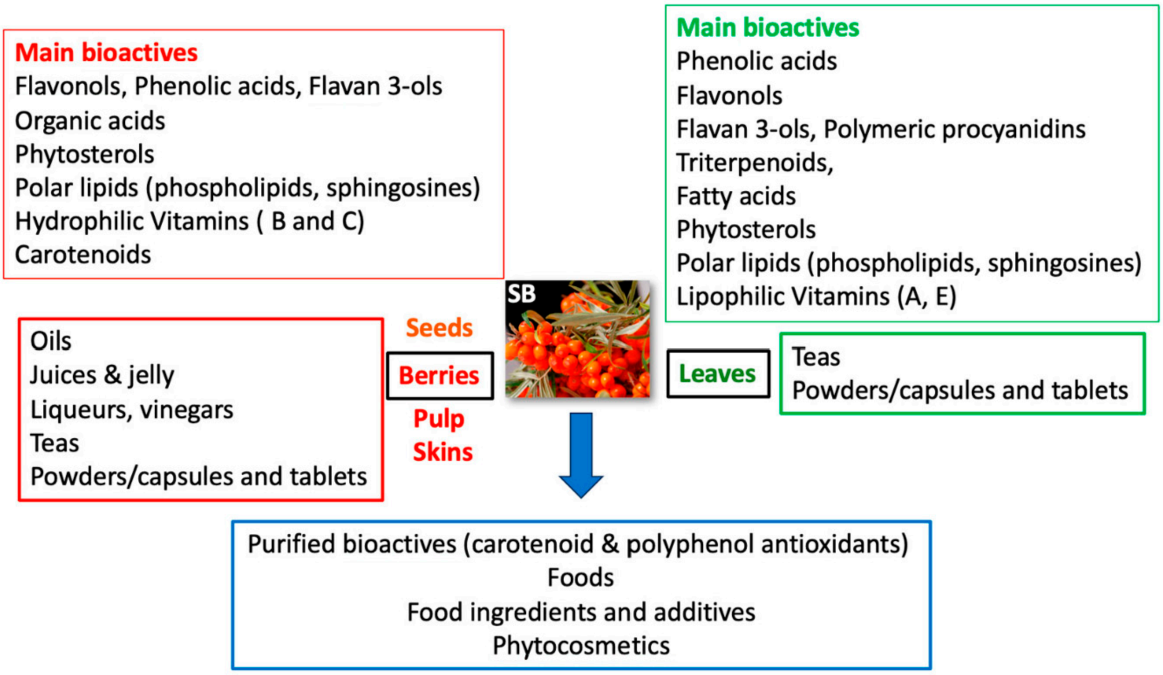

Supplementary Figure S1. The main chemical constituents and applications of sea buckthorn (*Hippophae rhamnoides* L. or *Elaeagnus rhamnoides* (L.) A. Nelson).
