# Supplementary material for: In Search of Authenticity Biomarkers in Food Supplements Containing Sea Buckthorn: A Metabolomics Approach
Source: Foods. 2023 Dec 15;12(24):4493. doi: 10.3390/foods12244493 (PMC10742966; doi:10.3390/foods12244493)
Supplement: Supplementary file 1 [file foods-12-04493-s001.zip › Supplementary Figure S2.pdf]

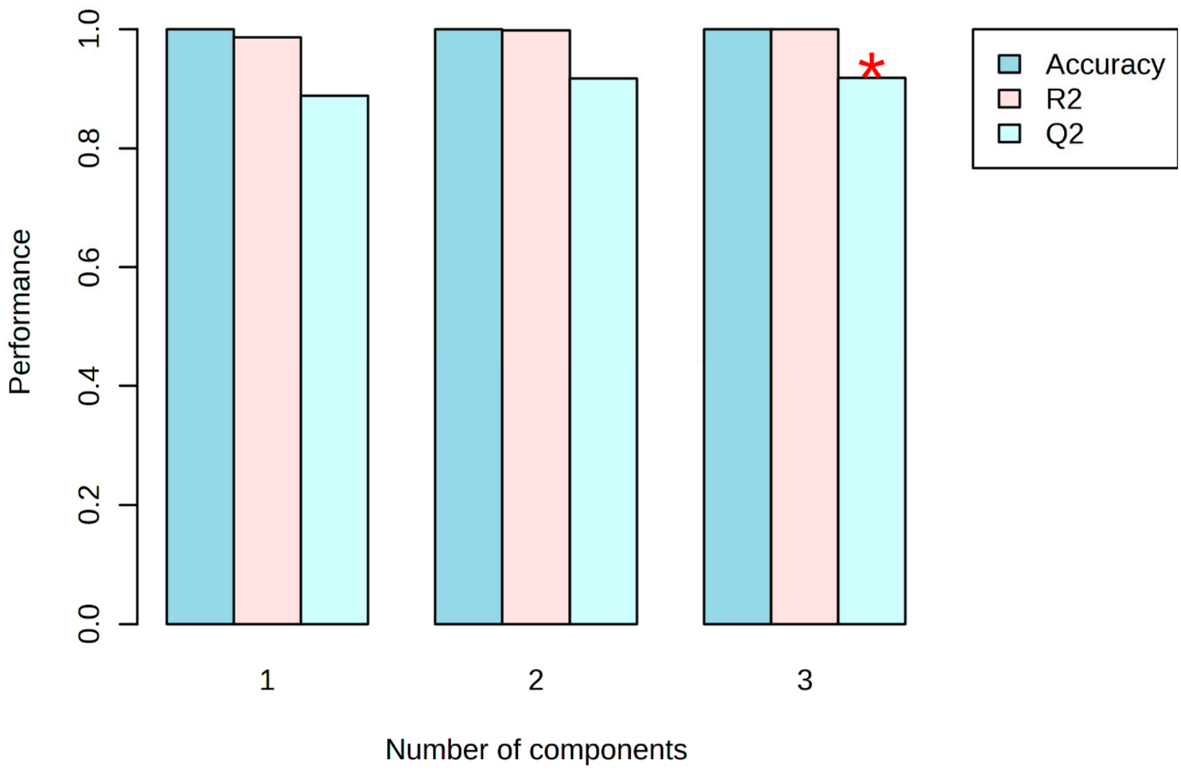

Supplementary Figure S2. Cross-validation graph showing the accuracy, R2, and Q2 values for the first three components, when comparing the composition of sea buckthorn leaves (L) and berries (B). For details see section 3.2.1.1 of the manuscript.
