# Supplementary material for: In Search of Authenticity Biomarkers in Food Supplements Containing Sea Buckthorn: A Metabolomics Approach
Source: Foods. 2023 Dec 15;12(24):4493. doi: 10.3390/foods12244493 (PMC10742966; doi:10.3390/foods12244493)
Supplement: Supplementary file 1 [file foods-12-04493-s001.zip › Supplementary Figure S3.pdf]

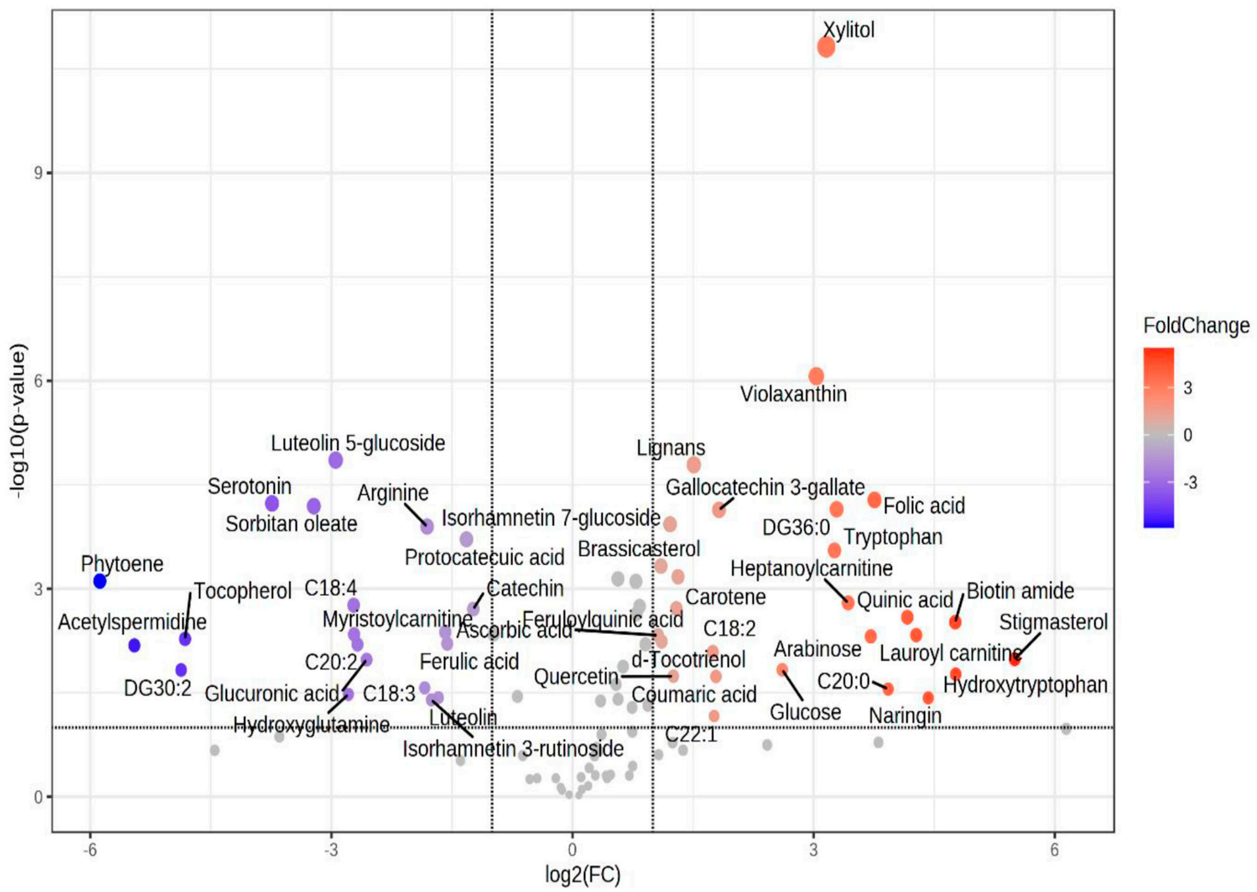

Supplementary Figure S3. Volcano plot algorithm used to determine log10(p-value) *versus* log2(FC) values and the dynamics (ncrease or decrease) of molecules′levels between SB leaves and berries.
