# Supplementary material for: In Search of Authenticity Biomarkers in Food Supplements Containing Sea Buckthorn: A Metabolomics Approach
Source: Foods. 2023 Dec 15;12(24):4493. doi: 10.3390/foods12244493 (PMC10742966; doi:10.3390/foods12244493)
Supplement: Supplementary file 1 [file foods-12-04493-s001.zip › Supplementary Table S1.pdf]

Supplementary Table S1. Identification of 98 molecules found in sea buckthorn leaves or berries, based on the MS data $[M+H]^+$ (*m/z* values). The experimental *m/z* values were compared with the average *m/z* values from the International database FooDB (https://foodb.ca/). The FOODB codes were mentioned, considering the accuracy of (theoretical – experimental) m/z values below 20 ppm.

$[M+H]^+$ (m/z)	Identification of putative biomarkers	FooDB code
151.0389	Arabinose	FDB001218
153.1304	Xylitol	FDB001134
155.0092	2,3-Dihydroxybenzoic (protocatecuic acid)acid	FDB012200
163.0778	L-4-Hydroxyglutamine	FDB000524
165.0587	p-Coumaric acid	FDB002593
169.0250	Vanillic acid	FDB000846
171.0317	Gallic acid	FDB000662
175.1362	Arginine	FDB002257
177.0098	Ascorbic acid	FDB001223
177.0581	Allantoic acid	FDB012646
177.1153	Serotonin	FDB012158
181.0491	Caffeic acid	FDB002558
181.1254	Glucose	FDB093715
183.1054	D-Glucitol	FDB011676
188.1777	N1-Acetylspermidine	FDB022530
193.0475	(-)-Quinic acid	FDB001170
195.0928	Ferulic acid	FDB012801
195.0775	D-Glucuronic acid	FDB006716
201.1098	Dodecanoic acid (lauric) C12:0	FDB003010
205.0913	L-Tryptophan	FDB002250
205.1836	Cubenene	FDB097255
216.1363	Propenoylcarnitine	FDB029297
221.0816	5-Hydroxy-L-tryptophan	FDB006231
229.1470	Myristic acid (C14:0)	FDB031009
244.1698	Biotin amide	FDB022636
255.2377	Palmitoleic acid (C16:1)	FDB004036
257.2542	Palmitic acid (C16:0)	FDB031084
266.1391	Thiamine	FDB008424
271.0951	Apigenin	FDB002798
273.0919	Naringenin	FDB000678
274.1814	Heptanoylcarnitine	FDB029350
275.1684	Glutaminylglutamine	FDB111844
275.2653	Digeranyl	FDB013789
277.2236	Coniferonic acid (C18:4)	FDB004289
279.2395	Linolenic acid (C18:3)	FDB012462
281.2551	Linoleic acid (C18:2)	FDB023610
283.231	Oleic acid (C18:1)	FDB031070
285.1744	*Cis*-Retinal	FDB023842

285.2967	Stearic acid (C18:0)	FDB031183
286.2192	Myristoylglycine	FDB029359
287.0621	Luteolin	FDB013255
287.0697	Kaempherol	FDB000633
291.0850	Catechin	FDB002571
300.2927	C18:1 Sphingosine	FDB021919
303.0011	Ellagic acid	FDB012575
303.0567	Quercetin	FDB011904
305.1810	Arachidonic acid (C20:4)	FDB011872
307.1251	Epigallocatechin	FDB017700
307.2564	Eicosatrienoic acid (C20:3)	FDB023082
309.2158	Eicosadienoic acid (C20:2)	FDB005834
311.2295	Gondoic (Eicosenoic) acid (C20:1)	FDB012639
313.2464	Arachidic acid (C20:0)	FDB004028
317.0697	Isorhamnetin	FDB000604
319.0358	Myricetin	FDB012724
333.1741	Carnosic acid	FDB014905
339.3240	Erucic acid C22:1	FDB004287
344.2458	Lauroyl carnitine	FDB022928
354.0253	Naringenin 5-sulfate	FDB093741
355.1131	kaempferol 3-rhamnoside-7-glucoside	FDB093501
357.1743	Ferulic acid 4-glucoside	FDB000256
369.1539	5-Feruloylquinic acid	FDB000247
372.3611	Myristoylcarnitine	FDB023615
377.1781	Riboflavin	FDB012160
397.3078	d-Tocotrienol	FDB001299
399.3222	Brassicasterol	FDB012496
400.3939	Palmitoyl carnitine	FDB021910
401.3795	Campesterol	FDB012521
413.3537	Stigmasterol	FDB001936
415.1521	Lignans	FDB005955
415.4086	Sitosterol	FDB007299
417.3843	Tocopherol	FDB002431
424.3788	Linoleyl carnitine	FDB023928
427.3821	alpha1-Sitosterol	FDB001304
428.3168	Stearoyl carnitine	FDB022278
429.2931	Sorbitan oleate	FDB001121
433.1588	Kaempferol 7-rhamnoside	FDB016474
435.1218	Naringenin 5-glucoside	FDB011867
442.1545	Folic acid	FDB014504
449.1252	Luteolin 5-glucoside	FDB006319
449.1281	Kaempferol 7-glucoside	FDB016495
459.0713	Gallocatechin 3-gallate	FDB021740
465.0979	Quercetin 7-glucoside	FDB002523
479.0677	Neoglucobrassicin	FDB017734

479.1042	Isorhamnetin 7-glucoside	FDB000606
537.4106	Carotene	FDB003842
537.4221	DG(30:2)	FDB024235
541.4506	DG(30:0)	FDB024289
545.5254	Phytoene	FDB015831
549.0808	NI	
569.4237	Zeaxanthin	FDB014726
581.1420	Naringin	FDB011866
597.1189	Quercetin 3-gluco-xyloside	FDB006895
601.4238	cis-Violaxanthin	FDB007098
611.1939	Quercetin 3-rutinoside	FDB002536
614.516	NI	
625.1908	Isorhamnetin 3-rutinoside	FDB000609
625.5710	DG(36:0)	FDB098471
627.1666	Quercetin 3,7-diglucoside	FDB016393
